# Comparative Characterization of Cells from the Various Compartments of the Human Umbilical Cord Shows that the Wharton’s Jelly Compartment Provides the Best Source of Clinically Utilizable Mesenchymal Stem Cells

**DOI:** 10.1371/journal.pone.0127992

**Published:** 2015-06-10

**Authors:** Arjunan Subramanian, Chui-Yee Fong, Arijit Biswas, Ariff Bongso

**Affiliations:** Department of Obstetrics and Gynaecology, Yong Loo Lin School of Medicine, National University Health System, National University of Singapore, Kent Ridge, Singapore, 119228, Singapore; University of Melbourne, AUSTRALIA

## Abstract

The human umbilical cord (UC) is an attractive source of mesenchymal stem cells (MSCs) with unique advantages over other MSC sources. They have been isolated from different compartments of the UC but there has been no rigorous comparison to identify the compartment with the best clinical utility. We compared the histology, fresh and cultured cell numbers, morphology, proliferation, viability, stemness characteristics and differentiation potential of cells from the amnion (AM), subamnion (SA), perivascular (PV), Wharton’s jelly (WJ) and mixed cord (MC) of five UCs. The WJ occupied the largest area in the UC from which 4.61 ± 0.57 x 10^6^ /cm fresh cells could be isolated without culture compared to AM, SA, PV and MC that required culture. The WJ and PV had significantly lesser CD40+ non-stem cell contaminants (26-27%) compared to SA, AM and MC (51-70%). Cells from all compartments were proliferative, expressed the typical MSC-CD, HLA, and ESC markers, telomerase, had normal karyotypes and differentiated into adipocyte, chondrocyte and osteocyte lineages. The cells from WJ showed significantly greater CD24+ and CD108+ numbers and fluorescence intensities that discriminate between MSCs and non-stem cell mesenchymal cells, were negative for the fibroblast-specific and activating-proteins (FSP, FAP) and showed greater osteogenic and chondrogenic differentiation potential compared to AM, SA, PV and MC. Cells from the WJ offer the best clinical utility as (i) they have less non-stem cell contaminants (ii) can be generated in large numbers with minimal culture avoiding changes in phenotype, (iii) their derivation is quick and easy to standardize, (iv) they are rich in stemness characteristics and (v) have high differentiation potential. Our results show that when isolating MSCs from the UC, the WJ should be the preferred compartment, and a standardized method of derivation must be used so as to make meaningful comparisons of data between research groups.

## Introduction

Mesenchymal stem cells have been derived from various sources. However, those of fetal origin face ethical issues as they are isolated from human abortuses while MSCs from adult bone marrow and organs have the disadvantages of painful invasive harvest, limited cell numbers, diminishing stemness properties with age and short-lived stemness properties *in vitro* [1,2]. These disadvantages have prompted interest in the exploration of other sources. Recently, primitive MSCs have been derived from various compartments of the human umbilical cord (UC) [3–8] and appear to be an attractive substitute.

The progressive expansion of the amniotic cavity between the 4^th^ and 8^th^ week of human embryonic development results in the formation of the tubular UC covered with the amniotic membrane and containing within it the yolk sac and allantois. Regression of the allantois and yolk sac occurs between the 6th and 8th weeks of gestation in the human. At term, the UC has an average length of 50–60 cm, mean diameter of 14.42 ± 1.50 mm and approximate weight of about 40g [9]. It contains two umbilical arteries and one umbilical vein embedded in the proteoglycan-rich gelatinous Wharton’s jelly (WJ) and surrounded by a single layer of amnion. Several groups have categorized the human UC into various compartments such as (i) the amniotic epithelial membrane (AM) (ii) subamnion or ‘cord lining’ (SA) (iii) intervascular Wharton’s jelly (WJ) and (iv) perivascular region (PV) surrounding the umbilical blood vessels [5,10].

MSCs have been isolated from each of these compartments by different authors [3–8]. At least six different methods of MSC derivation from these various compartments have been reported. Briefly, these methods include (i) cutting open tubular UC pieces, stripping out the umbilical blood vessels and scraping off or squeezing out the WJ with forceps from which stem cells are harvested [11,12], (ii) separation of the WJ without removing the umbilical blood vessels [13–17], (iii) culturing entire cord pieces with intact umbilical vessels as explants for a few days after which the cell outgrowths from the explants are separated and cultured as UC-MSCs (mixed cord, MC) [6,18–19], (iv) separation of the subamnion region (cord lining) with a razor blade, cutting it into small pieces and growing the pieces as explants from which the cell outgrowths are separated and cultured [7,20], (v) removal of the umbilical blood vessels, tying them at either end into loops and then placing the loops into an enzymatic solution to allow detachment of cells from the perivascular region which are then grown in culture [3] and (vi) cutting open cord pieces and placing the outer surface face down into an enzymatic solution to allow only the amniotic membrane cells to detach and then grow in culture [4,21–22].

The phenotypic profiles of the MSCs derived from these various compartments seem to be inconsistent across studies. Some authors have reported that the perivascular stem cells were positive for CD14, CD106 and CD117 [3,23–24] while others reported that they were negative [25]. Cord lining or subamnion MSCs were shown to be positive for CD34, CD45 and SOX2 in one study [26] and negative in another [27]. Similarly, the MSCs isolated from cultured whole UC pieces (MC) were shown to be positive for CD106 and CD117 in one report [28] and negative in another [29]. It has been reported that there is a differential distribution pattern of the various cytoskeletal proteins of stromal cells and extracellular matrix proteins in different zones of the SA, WJ and adventitia of the umbilical blood vessels [30]. Differences in differentiation potential have also been shown between stem cells of different compartments of the UC [31] and it was reported that a plethora of cellular sub-sources and populations can be derived from the human UC [32]. As such, some authors have suggested that the different regions of the cord be considered individually because the UC harbours a variety of embryonic or premature cell populations such as MSCs, endothelial stem/progenitor cells (EPCs, ECFCs) and HSCs (CD34 + and CD133+) [33].

In many publications authors do not describe precisely the exact compartment of the UC from which they derive their stem cells but simply use the terminology ‘human umbilical cord mesenchymal stem cells (UC-MSC)’ thus making comparison of results between groups ineffective with no standardization. Since umbilical cord MSCs are starting to reach the clinic, it is an urgent necessity to find out through rigorous comparative characterization whether differences exist between stem cell populations of the various compartments of the UC and identify which compartment generates the most clinically utilizable MSCs to allow global standardization of terminology and derivation methods so as to make comparison of data meaningful. We therefore undertook a rigorous study to comparatively evaluate the cell numbers, proliferation, viability, stemness properties and degree of differentiation potential of MSC populations between AM, SA, PV, WJ and MC to identify the compartment with the best clinical utility.

## Materials and Methods

### In situ histology of cross-sections of human umbilical cords

Written informed patient consent was received from the patients themselves and ethical approval was obtained from the Institutional Domain Specific Review Board (DSRB), Ministry of Health, Singapore to study the microanatomy and derive stem cell populations from human umbilical cords. Pieces (1–2 cm) of whole umbilical cords were fixed in 10% neutral buffered formalin (Sigma) for 24 h at room temperature and later embedded in paraffin. Cross-sections from the UC pieces were cut at 5 μm thickness using a Microtome (Leika, Wetzlar, Germany) and mounted on Superfrost Plus slides (Thermo Scientific, Waltham, MA, USA). The cross-sections were stained with Hematoxylin and Eosin (Sigma, MO) and photographed under bright field optics. A total of 10 cross-sectional areas per UC of the different regions were measured and compared in 5 different UCs. Detailed histology was studied at high magnification.

### Fresh live cell counts

The *in situ* cross-sections showed individual cells lying in the gelatinous matrix of the WJ while the cells of the other compartments (SA, AM, PV) were tightly held together by extracellular matrix (ECM) that required enzymatic digestion and primary cultures to harvest individual cells. As such, fresh live cell counts without culture could not be made for the SA and AM compartments. The fresh gelatinous WJ however was gently separated by aspiration with a syringe and 20G needle from cut-open 2 cm tubular pieces of UC, collected into tubes and mixed with equal volumes of culture medium comprised of DMEM-low glucose, fetal bovine serum (FBS) and antibiotic-antimycotic solution (Invitrogen Life Technologies, Carlsbad, CA). The WJ was resuspended in the medium by gentle pipetting and then centrifuged at 300 x g for 10 min. The supernatant was decanted and the cell pellet containing fresh Wharton’s jelly stem cells (hWJSCs) stained with 0.4%Trypan blue (vital dye) (Sigma Chemical Co, MO) for 1 min at room temperature. The number of live cells (unstained) was counted using a hemocytometer from 5 UCs and expressed as number of cells per cm of UC.

### Cell counts after culture

Each UC was cut into 5 equal pieces and cells isolated from each piece for the five compartments (AM, SA, WJ, PV and MC) using published established derivation protocols for each compartment [3–7,15,18]. The cells from the individual compartments were separately seeded into 100 mm tissue culture Petri dishes (BD, Franklin Lakes, NJ, USA) and grown in the same volumes of culture medium (DMEM-low glucose supplemented with L-glutamine, antibiotic-antimycotic solution). The Petri dishes were incubated at 37C in a 5% CO_2_ in air atmosphere until confluent primary cultures were established. The cells from all dishes were then disassociated with trypsin (TrypLE Express, Invitrogen Life Technologies, Carlsbad, CA) and the trypsinized cells then washed with similar volumes of medium. The washed cell pellets were seeded into fresh dishes and passaged to confluence. Serial passaging was carried out until the 10^th^ passage. Cell counts from individual compartments of five different UCs were determined in primary culture and cell morphology, proliferation and viability rates determined at the 3^rd^, 5^th^ and 10^th^ passages.

### Cell morphology

The morphological changes during culture (primary and passages) were monitored and photographed using inverted phase contrast optics (Nikon Instruments, Tokyo, Japan).

### Cell proliferation

The cell proliferation rates were carried out using a BrdU cell proliferation assay kit (Cell Signaling, Massachusetts, USA) according to the manufacturer’s instructions. Briefly, the cells were incubated for 30 min with the BrdU solution (10μM) followed by replacement of the solution with fixing/denaturing solution and further incubation for another 30 min. The cells were then incubated for 1 h with BrdU detection antibody solution followed by three washes with the wash buffer. The HRP-conjugate solution was then added to the cells and incubation carried out for 30 min followed by similar washing steps and further incubation with the substrate solution for 30 min. The enzymatic reaction was finally stopped and quantified by measuring the absorbance at 450nm using a microplate ELISA reader (μQuant-BioTek, Winooski, VT).

### Cell viability

The cell viability assay was performed using MTT reagent kit [3-(4, 5-dimethylthiazolyl-2)-2, 5-diphenyltetrazolium bromide] (Sigma) according to the manufacturer’s instructions. Briefly, 10 μl MTT reagent (final concentration of 0.5 mg/ml) was added into each well and the culture dishes incubated for 4 h until a purple precipitate was visible. The supernatant was then aspirated and 100 μl of the detergent reagent from the kit was added to each well and dishes incubated in the dark for 2 h. Absorbance at 570 nm was spectrophotometrically measured using a microplate ELISA reader (μQuant-BioTek, Winooski, VT) with a reference wavelength of 630 nm.

### CD marker analysis

Briefly, cell monolayers derived from each compartment of the UC were dissociated using trypsin (TrypLE Express, Invitrogen) for 3–5 min. The cells were then washed in phosphate buffered saline without calcium and magnesium (PBS ^(-)^) and then blocked with 10% normal goat serum (NGS) (Invitrogen) to prevent non-specific binding. The cells were then incubated with mouse monoclonal primary antibodies for a series of CD markers viz., CD14, CD19, CD24, CD29, CD34, CD40, CD44, CD45, CD49d, CD73, CD90, CD105, CD108, CD117, CD140b, CD146, CD271, HLA-ABC and HLA.DR (1:100, Biolegend, San Diego, CA) for 30 min. This was followed by incubation with Alexa Fluor 488 (1:750) goat anti-mouse secondary antibody (Invitrogen Life Technologies, Carlsbad, CA) for 30 min. The cells were finally washed in PBS ^(-)^, re-suspended in 10% NGS and filtered using a 70 μm nylon strainer (BD Bioscience) to remove any cell clumps and analyzed using a CyAn ADP Analyzer (Beckman Coulter, Fullerton, CA).

### Telomerase analysis (TRAP assay)

The telomerase activity of the cells derived from each compartment of the UC was analyzed using the TRAPEZE RT Kit (Chemicon, Millipore, Temecula, CA, USA) according to the manufacturer’s protocols. Briefly, the cells were first lysed in CHAPS buffer on ice for 30 min and the cell lysates then centrifuged at 12,000 x g for 20 min at 4°C. The supernatant was decanted and the cell lysate analyzed for protein concentration. From each sample 800 ng of protein was used for the TRAP assay. The positive control was TSR8 that came with the kit where telomerase activity was expressed as total-generated-product (TGP) unit. The logarithmic plot of the TSR8 standard curve was plotted by net fluorescence against the corresponding TGP unit of the positive control (TSR8). The TGP units of each sample were obtained from the standard curve by using the relative net fluorescence increase as according to the kit procedure and a RT-PCR-based Telomere Repeat Amplification Assay (TRAP) assay was carried out using a qRT-PCR machine (Applied Biosystems).

### Pluripotent marker analysis

The presence of ESC markers in the cells of each compartment was determined using immunohistochemistry (IHC) and immunocytochemistry (ICC). For IHC, histological sections (5 μm) of UC pieces mounted on Superfrost Plus slides were used and for ICC, fixed cells from each region of the UC were analyzed. The histological sections and fixed cells were washed twice with PBS and blocked with 10% NGS for 20–30 min. The sections and cells were then incubated with rat primary monoclonal antibody SSEA-3 and mouse monoclonal primary antibodies SSEA-4, Tra-1-60, Tra-1-81 (1:100, Millipore) overnight at 4°C. This was followed by incubation with goat anti-rat and goat anti-mouse secondary antibodies (Alexa Fluor, Invitrogen) for 1 h. The cells and sections were then washed with PBS and stained with 4'-6-diamidino-2-phenylindole (DAPI; 0.5μg/ml) (Invitrogen) for 5 min at room temperature. Finally the sections and cells were washed once with PBS, examined and photographed using a confocal microscope.

### Genomic markers

The presence of pluripotent and fibroblast markers in the cells of each compartment was determined using the quantitative real time polymerase chain reaction (qRT-PCR). Total RNA from the cells of each compartment of the UC was extracted using TRIzo reagent (Invitrogen) and its quality and quantity measured using a Nanodrop spectrophotometer (Nanodrop technologies, Wilmington, DW). All RNA samples were treated with DNase-I prior to first strand cDNA synthesis with random hexamers using the SuperScript first strand synthesis system (Invitrogen). Primer sequences were taken from earlier published studies and are summarized in Table 1. qRT-PCR analysis was performed with the ABI 7500 Fast Real-Time PCR System (Applied Biosystems, Foster City, CA) using SYBR green as previously described [34] and relative quantification was performed using the comparative CT (2-ΔΔCT) method.

**Table 1:** Gene and primer sequences used in this study.

Gene	Primer Sequence
OCT1	F: 5’- GCAACCCTGTTAGCTTGGTC -3’
	R: 5’- CTCTCCTTTGCCCTCACAAC -3’
OCT2	F: 5’- AGGCCTCAGCGTTCTCTTTT -3’
	R: 5’- TGCCAGTCCCTTCTCTCTTC -3’
OCT4A	F: 5’- AGTGAGAGGCAACCTGGAGA -3’
	R: 5’- GTGAAGTGAGGGCTCCCATA -3’
OCT4B	F: 5’- TATGGGAGCCCTCACTTCAC -3’
	R: 5’- CAAAAACCCTGGCACAAACT -3’
NANOG	F: 5’- TGAACCTCAGCTACAAACAG -3’
	R: 5’- TGGTGGTAGGAAGAGTAAAG-3’
SOX2	F: 5’- AGCTACAGCATGATGCAGGA -3’
	R: 5’- GGTCATGGAGTTGTACTGCA -3’
FAP	F: 5’- TCAACTGTGATGGCAAGAGCA -3’
	R: 5’- TAGGAAGTGGGTCATGTGGGT -3’
FSP	F: 5’- GATGAGCAACTTGGACAGCA -3’
	R: 5’- CTTCCTGGGCTGCTTATCTG -3’
PPAR γ	F: 5'-GGCTTCATGACAAGGGAGTTTC-3'
	R: 5’- AACTCAAACTTGGGCTCCATAAAG -3’
FABP4	F: 5’- TTGACGAAGTCACTGCAGATGA -3’
	R: 5’- CAGGACACCCCCATCTAAGGT -3’
PREF-1	F: 5’- TACGAGTGTCTGTGCAAGC -3’
	R: 5’- ACACAAGAGATAGCGAACACC -3’
CEBP β	F: 5’- ACTTCAGCCCGTACCTGGAG -3’
	R: 5’- GAGAAGAGGTCGGAGAGGAAGT -3’
OCN	F: 5’- CCCAGGCGCTACCTGTATCAA -3’
	R: 5’- GGTCAGCCAACTCGTCACAGTC -3’
OPN	F: 5’- ACAGCCACAAGCAGTCCAGATT -3’
	R: 5’- TGCTCATTGCTCTCATCATTGG -3’
ALP	F: 5’- GGACCATTCCCACGTCTTCAC -3’
	R: 5’- CCTTGTAGCCAGGCCCATTG -3’
BSP	F: 5’- CCAGAGGAAGCAATCACCAAA -3’
	R: 5’- TTGAGAAAGCACAGGCCATTC -3’
COL2A1	F: 5’- GTGACAAAGGAGAGGCTGGA -3’
	R: 5’- ACCTCTAGGGCCAGAAGGAC -3’
COMP	F: 5’- GGAGATCGTGCAGACAATGA -3’
	R: 5’- GAGCTGTCCTGGTAGCCAAA -3’
FMOD	F: 5’- CAACCAGCTGCAGAAGATCC -3’
	R: 5’- CAGAAGCTGCTGATGGAGAA -3’
SOX9	F: 5’- GTACCCGCACTTGCACAAC -3’
	R: 5’- GTAATCCGGGTGGTCCTTCT -3’
GAPDH	F: 5’- GCACCGTCAAGGCTGAGAAC -3’
	R: 5’- GGATCTCGCTCCTGGAAGATG -3’

### Cell differentiation

For adipocyte differentiation, cells from the various compartments were seeded into 6-well tissue culture dishes with an initial seeding density of 10 x 10^4^ cells/dish and incubated at 37°C in a 5% CO_2_ atmosphere for 24 h to allow attachment and the medium was then changed to adipogenic induction medium contain DMEM-Low glucose supplemented with 10% FBS, 1% penicillin/streptomycin, 0.01 mg/ml insulin (Invitrogen), 1 μM dexamethasone, 0.5mM 3-isobutyl-1-methyl-xanthine (IBMX) and 0.2 mM indomethacin (Sigma). The cells were maintained for 28 days in the induction medium with medium changes every 48 h. The cells were then stained with Oil Red O (Sigma). Briefly, the cells were first fixed with 4% paraformaldehyde for 10 min and then with 60% isopropanol for 5 min after rinsing with PBS. They were then stained with Oil Red O for 5 min, rinsed with distilled water until the water was clear and counter-stained with hematoxylin. The stained cells were visualized using a phase contrast microscope (Nikon Instruments, Tokyo, Japan).

For osteogenic differentiation, cells from the various compartments were seeded (10 x 10^4^ cells/dish) into 6-well tissue culture dishes and incubated at 37°C in a 5% CO_2_ for 24 h to allow attachment and the medium then switched to osteogenic medium contain DMEM supplemented 5% FBS, 0.17mM L-ascorbic-acid, 100nM dexamethasone, 1% penicillin/streptomycin and 10mM β-glycerophosphate (Sigma) and the cells cultured for 28 days with fresh changes of medium every 48 h. The cells were then subjected to Von Kossa staining. Briefly, the cells were rinsed with PBS and fixed in 3.7% formaldehyde solution for 10 min at room temperature. They were then washed with distilled water and stained in 1% silver nitrate solution under UV light for 60 min. The cells were then washed again with distilled water, treated with 3% sodium thiosulfate for 5 min and counter-stained with 1% Nuclear Fast Red (Sigma) for 5 min. The stained cells were washed with distilled water and photographed under inverted phase contrast optics (Nikon).

For chondrogenic differentiation, cells from the various compartments were seeded (10 x 10^4^ cells/dish) into 6-well tissue culture dishes and incubated at 37°C in a 5% CO_2_ for 24 h to allow attachment and the medium was then changed to chondrogenic medium contain DMEM (4.5 g/l glucose) supplemented with 1% penicillin/streptomycin, 1% ITS, 0.17mM L-ascorbic-acid, 100nM dexamethasone, 1mM sodium pyruvate, 0.35 mM proline and 10 ng/ml TGFß-3 (Sigma) and the cells cultured for 28 days with fresh changes of medium every 48 h. The cells were then stained with Alcian blue (Sigma). Briefly, the cells were disassociated with trypsin (Invitrogen), washed with PBS (-) and cytospun directly on to glass slides using Cyotospin (Thermo Scientific, Barrington, IL) at 500 rpm for 5 min. The cytospun slides were stained with 0.5% Alcian Blue (Sigma, St. Louis, MO) for 30 min at room temperature and then rinsed with tap water. The slides were counter-stained with 0.1% Nuclear Fast Red (Sigma) for 5 min and photographed under inverted phase contrast optics (Nikon).

### Degree of differentiation

The degree of adipocyte, osteogenic and chondrogenic differentiation for each compartment was evaluated quantitatively using the protocol of Mennan et al [35]. Scores were attributed to the number of cells stained and staining intensity in adipocyte, osteocyte and chondrocyte-stained slides and then subjected to statistical analysis. The degree of differentiation was also evaluated from gene expression levels for the adipocyte, osteocyte and chondrocyte genomic markers using a scoring system ranging from 0 to 5 (+++++).

### Statistical analysis

All cell counts and stemness assays were analyzed using one-way ANOVA with Bonferroni’s multiple comparison post hoc analysis using the statistical package for Social Sciences (SPSS 13). The results were expressed as mean ± SEM from three different replicates for individual assays and a value of p<0.05 was considered to be statistically significant. The degree of differentiation into the adipocyte, osteocyte and chondrocyte lineages was calculated using the Kruskall-Wallace and Bonferri post hoc tests.

## Results

### Histology

**Fig 1:**
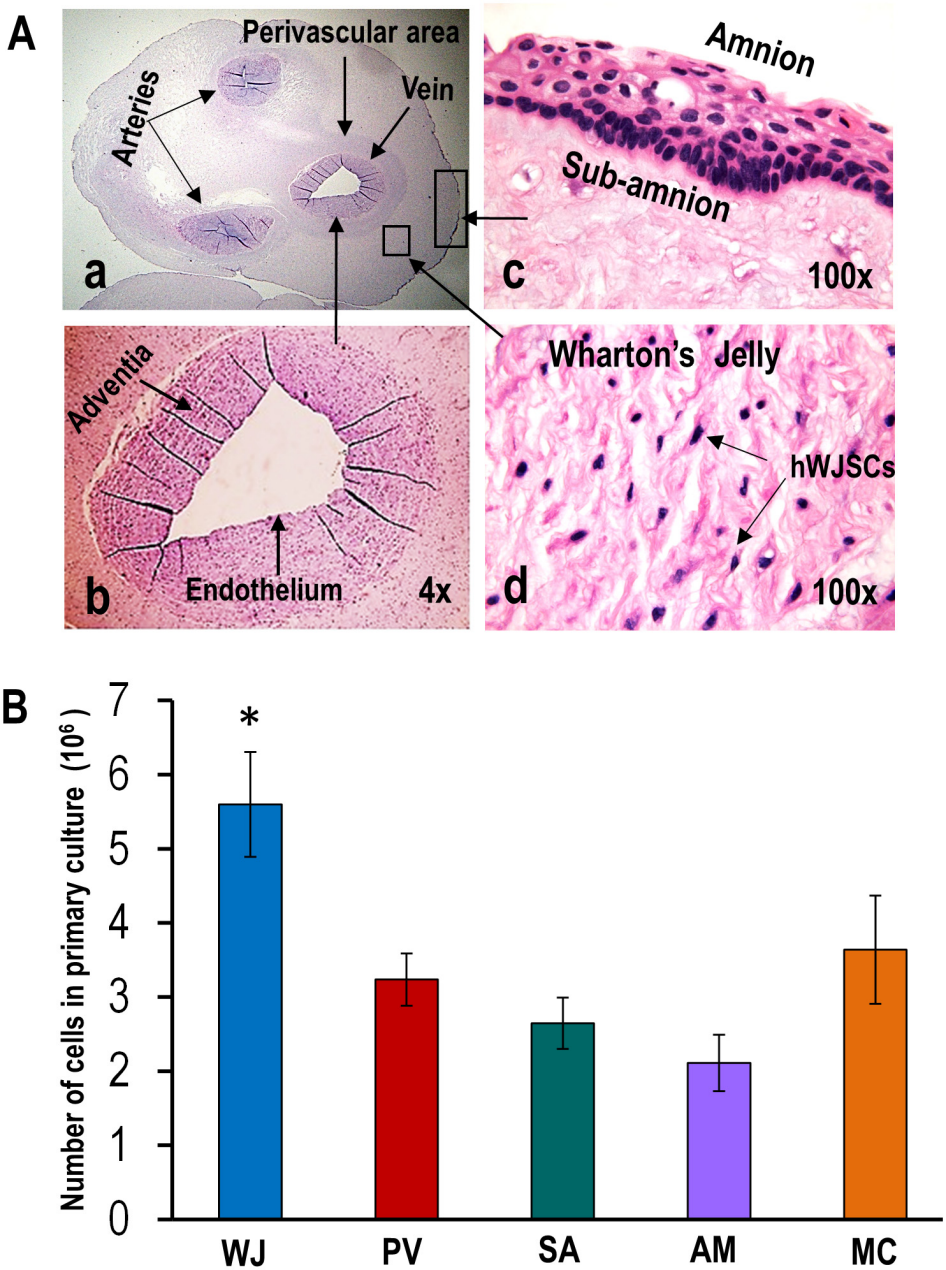
(A) (a-d) In situ H & E histological cross-sections of the human umbilical cord showing the various regions [Wharton’s jelly (WJ), perivascular area (PV), subamnion (SA), amnion (AM)] from which MSCs were derived. Note individual MSCs (hWJSCs) lying in gelatinous matrix of WJ. **(B)** Histogram showing significantly greater numbers of MSCs (hWJSCs) in the WJ in primary culture compared to PV, SA, AM and mixed cord cultures (MC) (*p<0.05).

In situ histological H & E cross-sections consistently showed clear-cut definite regions in all five UCs. Starting from the outside, the compartments could be identified as the amnion (AM), subamnion (SA), Wharton’s jelly (WJ) (in which lied two arteries and a vein) around which were thin perivascular areas (PV) (Fig 1Aa-d). The AM and SA were tightly attached to each other requiring enzymatic manipulation and culture for cell isolation. The intervascular WJ region was a gelatinous matrix occupying the largest area and volume of the UC. Large numbers of individual cells with a stellate morphology could be seen lying within the WJ matrix that could be isolated without enzymatic manipulation and culture. To prevent contamination with WJ cells, PV cells could be separated from around the blood vessels only by enzymatic manipulation after stripping out the blood vessels from the rest of the UC (Fig 1Aa-d). Since the WJ cells were lying loosely within the WJ they were the easiest to isolate without enzymatic treatment and culture.

### Cell counts before and after culture

Approximately, 4.61 ± 0.57 x 10^6^ fresh live cells without culture were isolated per cm of human UC from the WJ. Such live cells could not be obtained from the other compartments (PV, SA, AM, MC) without primary culture. In primary culture, cells from WJ produced monolayers faster with significantly greater cell numbers compared to the PV, SA, AM and MC (p<0.05) (Fig 1B).

### Cell morphology

**Fig 2:**
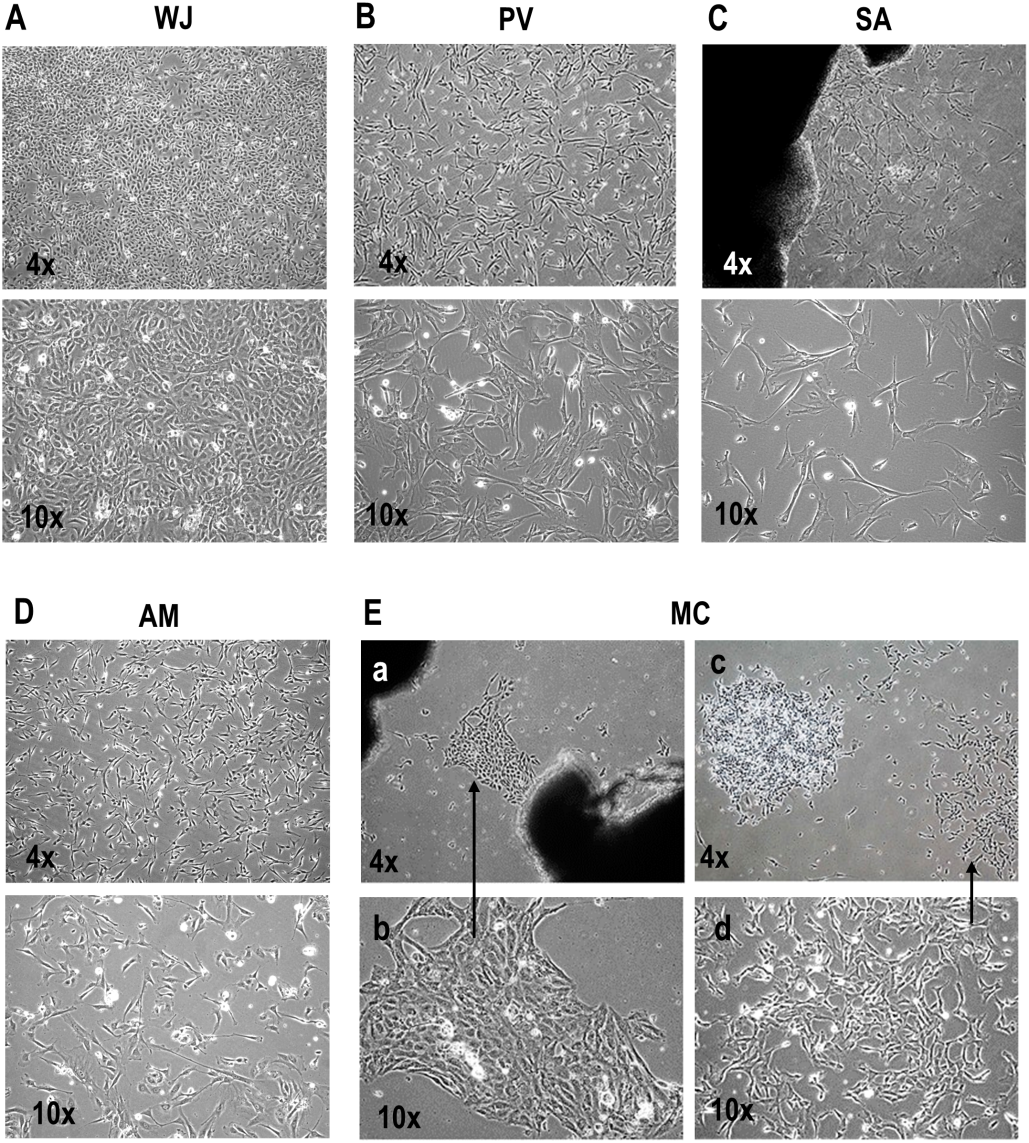
(A-E) Morphology of MSCs derived from WJ, PV, SA, AM and MC in primary culture. **(A-D)** The MSCs from WJ showed one type of stellate epithelioid-like morphology while those from the PV, SA and AM showed one type of short fibroblast-like morphology. **(E)** The MSCs from MC cultures showed cell islands with two types of morphology **(a-b)** epithelioid-like and **(c-d)** short fibroblast-like.

MSCs derived from the various compartments showed different morphologies in primary culture. The hWJSCs grew as confluent monolayers with one type of stellate morphology only (Fig 2A) while the cells from the PV, SA and AM showed short fibroblast-like cell morphology in primary culture (Fig 2B–2D). Primary explants of MC cultures showed islands of cells with two distinct types of morphology (epithelioid-like) (Fig 2Ea-b) and short fibroblast-like (Fig 2Ec-d).

### Cell Proliferation (BrDU) and Cell viability (MTT)

**Fig 3:**
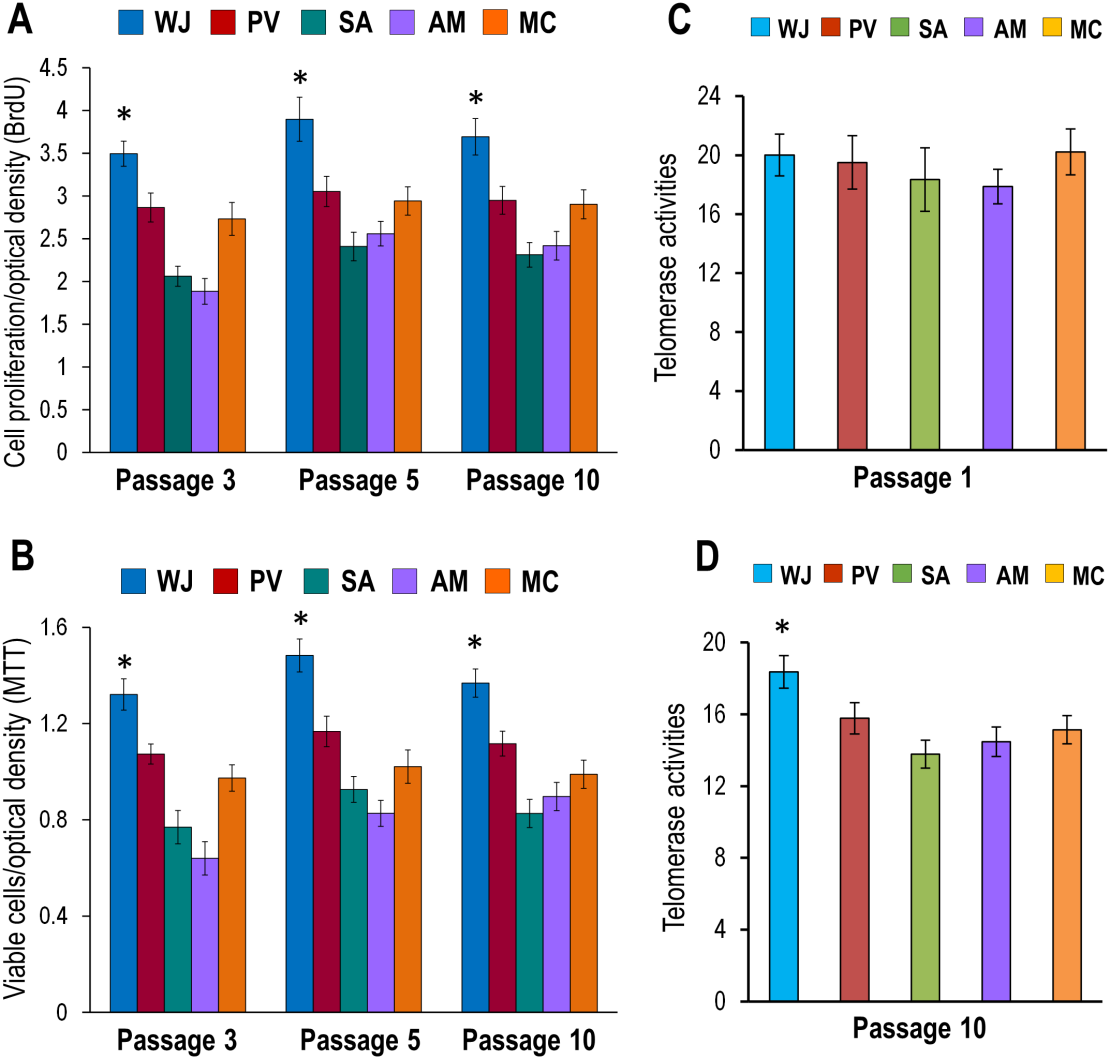
(A-D) Cell proliferation, viability and telomerase levels of MSCs derived from WJ, PV, SA, AM and MC. **(A)** BrdU assays showed significantly greater proliferation rates at passages 3, 5 and 10 in cells from WJ compared to PV, SA, AM and MC (*p<0.05). **(B)** MTT assays showed significantly greater cell viability at passages 3, 5 and 10 in cells from WJ compared PV, SA, AM and MC (*p<0.05). **(C)** qRT-PCR analysis for telomerase showed similar levels of telomerase activity with no significant differences (*p*>0.05) in early passages (P1) for cells derived from WJ, PV, SA, AM and MC but **(D)** at late passages (P10) the telomerase levels for WJ were significantly greater than PV, SA, AM and MC (*p<0.05).

The cells from WJ showed significantly greater cell proliferation and viability rates compared to cells from the PV, SA, AM and MC (p<0.05) at passages 3, 5 and 10 (Fig 3A and 3B). The proliferation rate of cells from WJ increased from 17.98 ± 2.11% to 48.97 ± 3.58% (P3), 20.33 ± 1.55% to 34.76 ± 2.76% (P5) and 20.15 ± 1.21% to 37.40 ± 1.89% (P10) while viability rates increased from 18.76 ± 2.09% to 51.56 ± 4.17% (P3), 21.31 ± 1.16% to 44.27 ± 2.99% (P5) and 18.39 ± 1.04% to 39.61 ± 3.01% (P10).

### Telomerase analysis

qRT-PCR analysis showed similar levels of telomerase activity at passage 1 (P1) for cells from the PV, SA, AM and MC but at passage 10 (P10) the levels remained high in cells from WJ and were significantly greater than those from PV, SA, AM and MC (p<0.05) (Fig 3C and 3D).

### CD marker analysis

**Fig 4:**
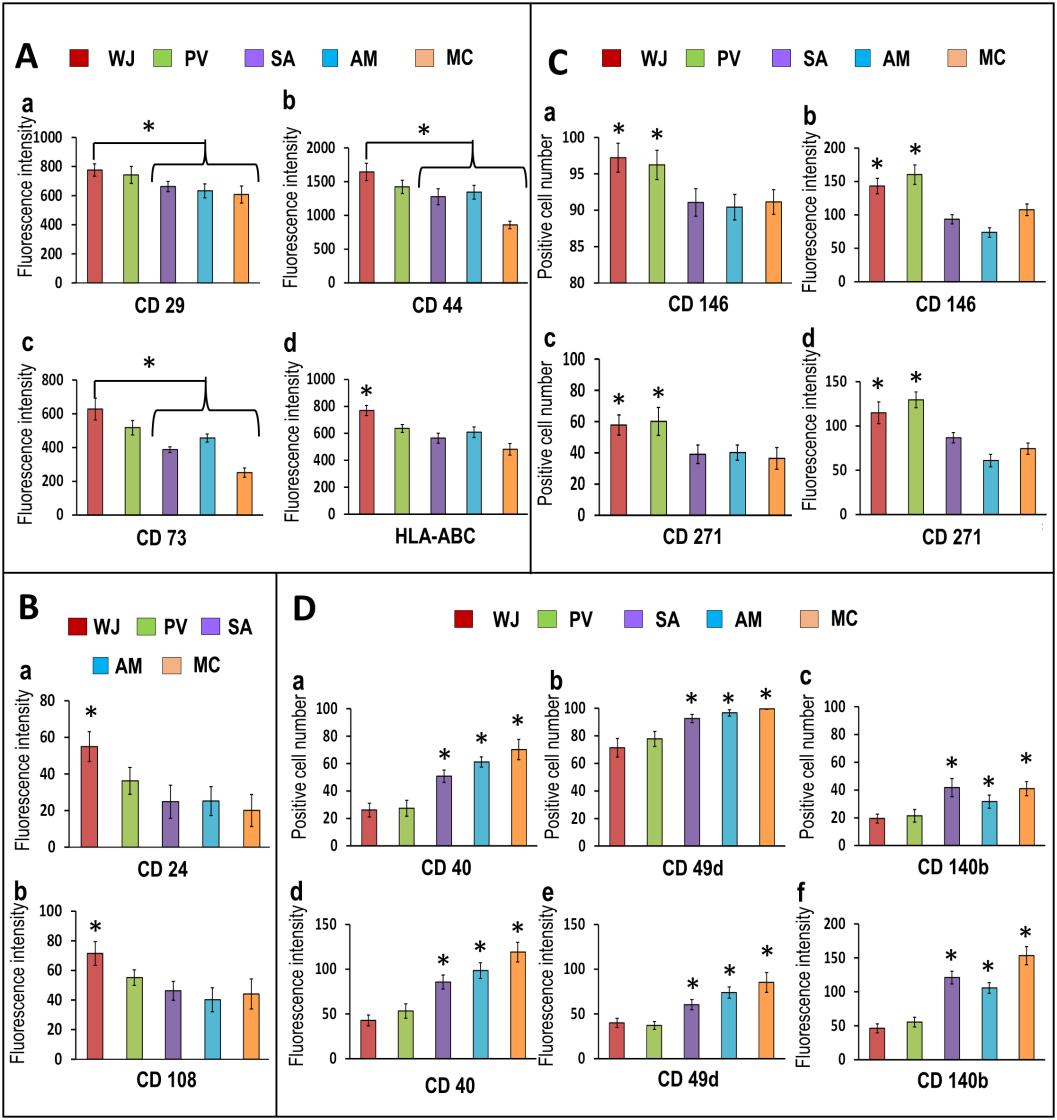
(A-D) CD marker analysis of cells derived from WJ, PV, SA, AM and MC. **(A) (a-d)** and **(B) (a-b)** The mean fluorescence intensities were significantly greater in cells from WJ compared to PV, SA, AM and MC for CD29, CD44, CD73 and HLA-ABC and for CD24 and CD108 (*p<0.05). **(C)** The percentages of positive cells **(a,c)** and mean fluorescence intensities **(b,d)** for CD146 and CD271 were significantly greater in cells from WJ and PV compared to SA, AM and MC (*p<0.05). **(D)** SA, AM and MC showed significantly greater percentages of positive cells **(a,b,c)** and mean fluorescence intensities **(d,e,f)** for the fibroblast markers CD40, CD49d and CD140b compared WJ and PV (*p<0.05).

The cell populations derived from all regions of the UC (WJ, PV, SA, AM and MC) were positive for the MSC-CD signature markers CD29, CD44, CD73, CD90, CD105 and HLA-ABC (97.81 ± 0.37% to 99.50 ± 0.11%) and negative for CD14, CD19, CD34, CD45, CD117 and HLA-DR ABC (3.73 ± 0.26% to 22.43 ± 0.46%) with no statistically significant differences in the percentages between the markers for the various regions. However, the mean ± SEM fluorescence intensities for CD29, CD44, CD73 and HLA-ABC were significantly greater in cells from WJ (627.91 ± 64.16 to 1643.19 ± 127.46) compared to cells from PV, SA, AM and MC (251.19 ± 27.20 to 1343.17 ± 103.11) (Fig 4Aa-d).

CD24 and CD108 were reported to reliably discriminate between MSC and non-stem cell mesenchymal cell cultures with significantly increased expression in MSCs [36]. In the present study, the mean ± SEM fluorescence intensities for CD24 and CD108 were significantly greater in cells in WJ (54.95 ± 8.16 to 71.46 ± 8.03) compared to the cells from PV, SA, AM and MC (Fig 4Ba-b).

WJ and PV showed significantly greater percentages of cells that were positive for the typical pericyte markers CD146 and CD271 (57.79 ± 6.47% to 97.21 ± 1.98%) with significantly greater mean ± SEM fluorescence intensities (143.09 ± 11.60 to 201.48 ± 13.27) compared to SA, AM and MC (p<0.05) (Fig 4Ca-d).

CD49d and CD140b are markers that are not consistently expressed by MSCs [37–39]. The SA, AM and MC cell populations showed significantly greater percentages of positive cells and mean fluorescence intensities for these two markers compared to WJ and PV (p<0.05). CD40 has been reported to be significantly increased in non-stem MSC cultures compared with cultures rich in MSCs [36] and numerous reports have identified CD40 expression amongst non-stem cell contaminants such as endothelial cells, smooth muscle cells, fibroblasts and epithelial cells [37–39]. In the present study, CD40+ cell populations were significantly greater for SA (50.77%), AM (61.16%), and MC (70.22%) compared to WJ (26.12%) and PV (27.42%) (p<0.05) (Fig 4Da-f).

### Pluripotency and fibroblast markers

**Fig 5:**
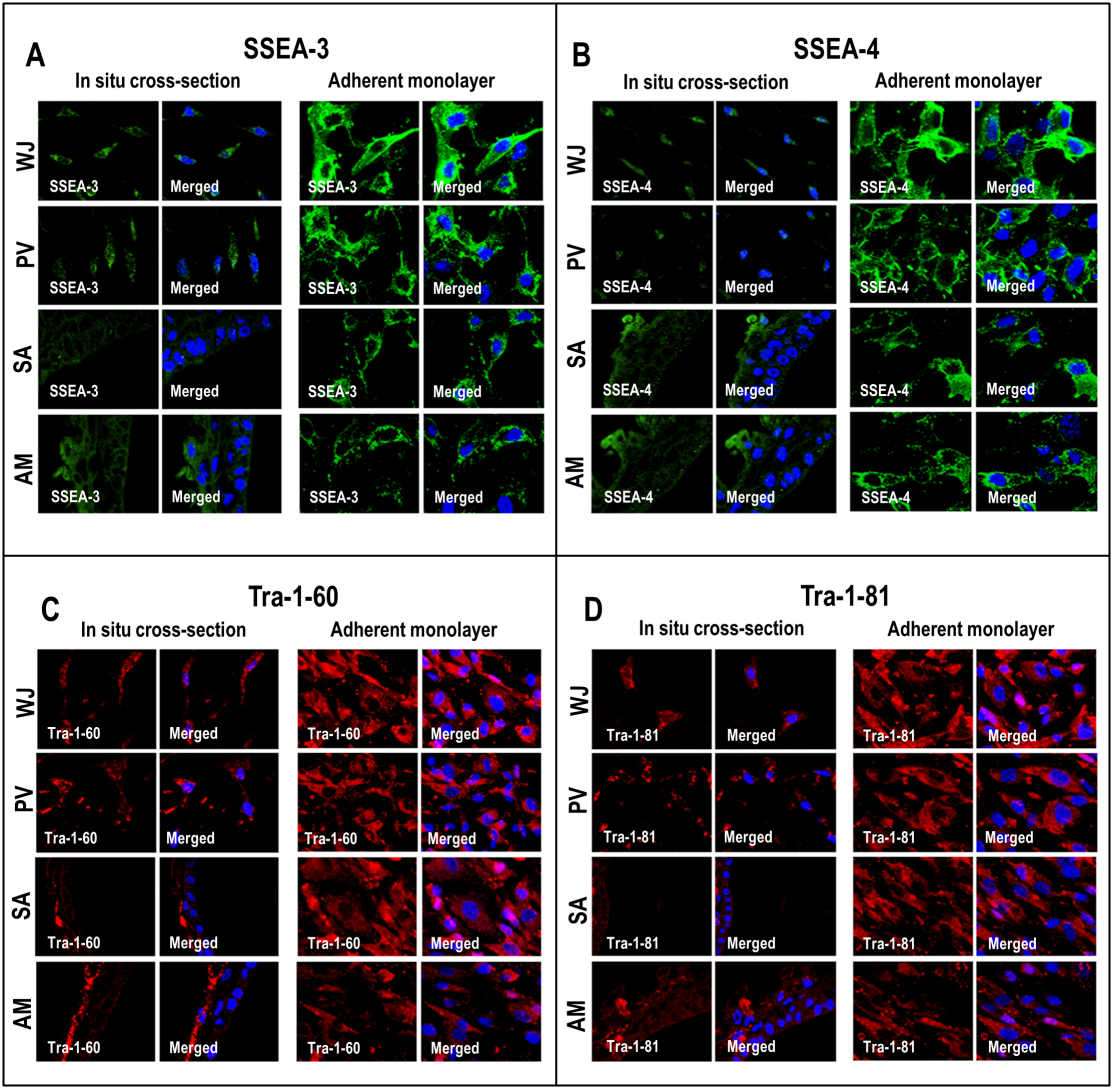
(A-D) Confocal microscopic analysis of embryonic stem cell (ESC) markers in cells of *in situ* cross-sections and adherent monolayers. WJ, PV, SA and AM were positive for SSEA-3, SSEA-4, Tra-1-60 and Tra-1-81.

**Fig 6:**
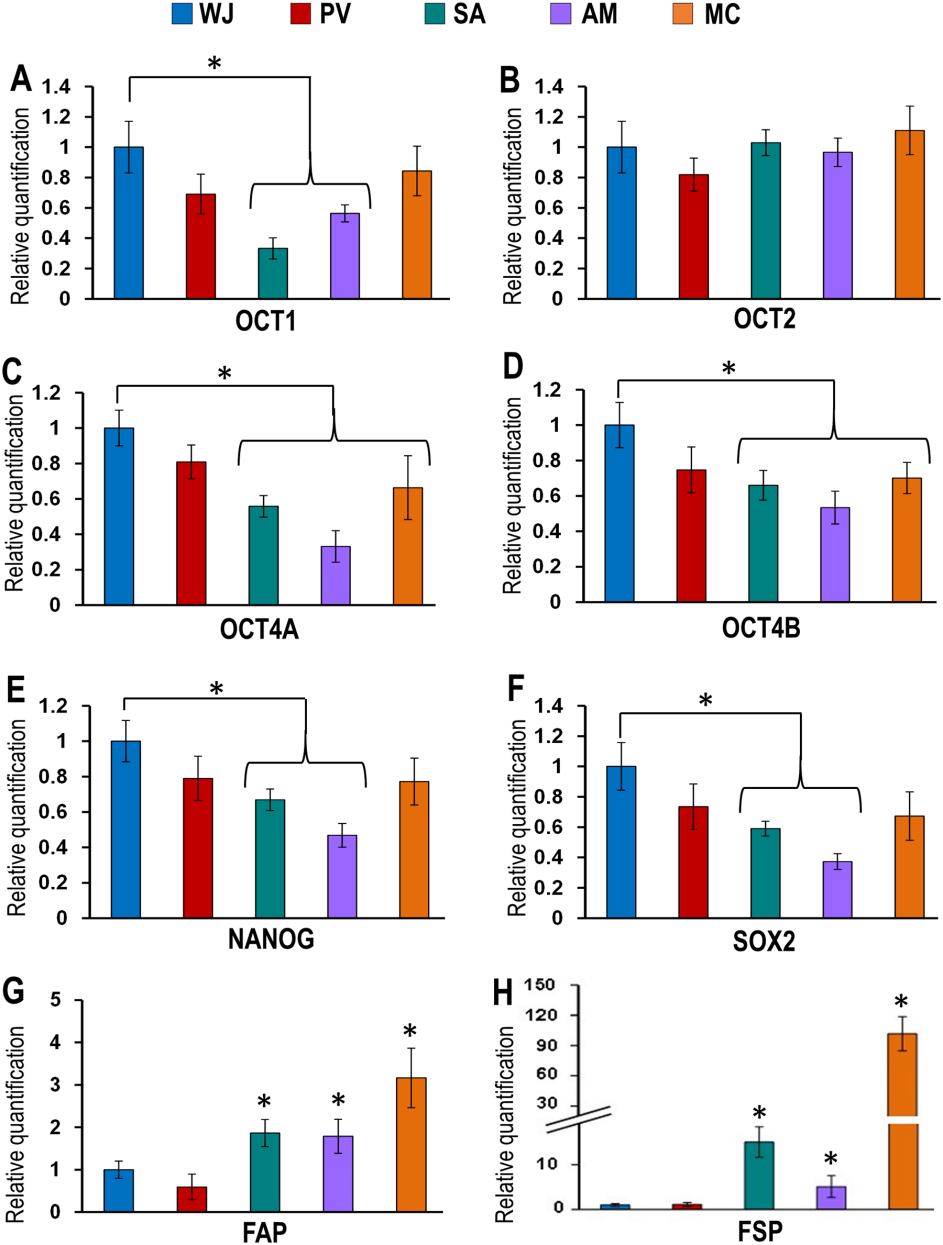
(A-F) qRT-PCR for embryonic stem cell (ESC) pluripotent genomic markers in cells derived from the WJ, PV, SA, AM and MC. Cells derived from WJ expressed significantly greater levels of the pluripotency genes OCT1, OCT4A, OCT4B, NANOG and SOX2 compared to cells from SA, AM and MC. (*p<0.05). **(G-H)** Cells from SA, AM and MC showed significantly greater levels of expression of the fibroblast-related genes FAP and FSP compared to WJ and PV (*p<0.05).

Immunohistochemical staining of cells from the various compartments of the UC were all positive for the pluripotency markers SSEA-3, SSEA-4, Tra-1-60 and Tra-1-81 (Fig 5A–5D). qRT-PCR analysis showed that the cells from all the compartments also expressed the pluripotency genes OCT1, OCT2, OCT4A, OCT4B, NANOG and SOX2 (Fig 6A–6F). However, the expression levels for OCT1, OCT4A, OCT4B, NANOG and SOX2 pluripotent genes were significantly lower for cells from the SA and AM compared to WJ (Fig 6A and 6C–6F). Also, OCT4A and OCT4B genes were significantly lower for cells from the MC compared to WJ (Fig 6C and 6D). The cells from the SA, AM and MC showed significantly high expression levels of the fibroblast-related genes such as fibroblast activating protein (FAP) and fibroblast stimulating protein (FSP) (0.86 fold to 100.64 fold) compared to cells from the WJ and PV (Fig 6G and 6H).

### Differentiation

**Fig 7:**
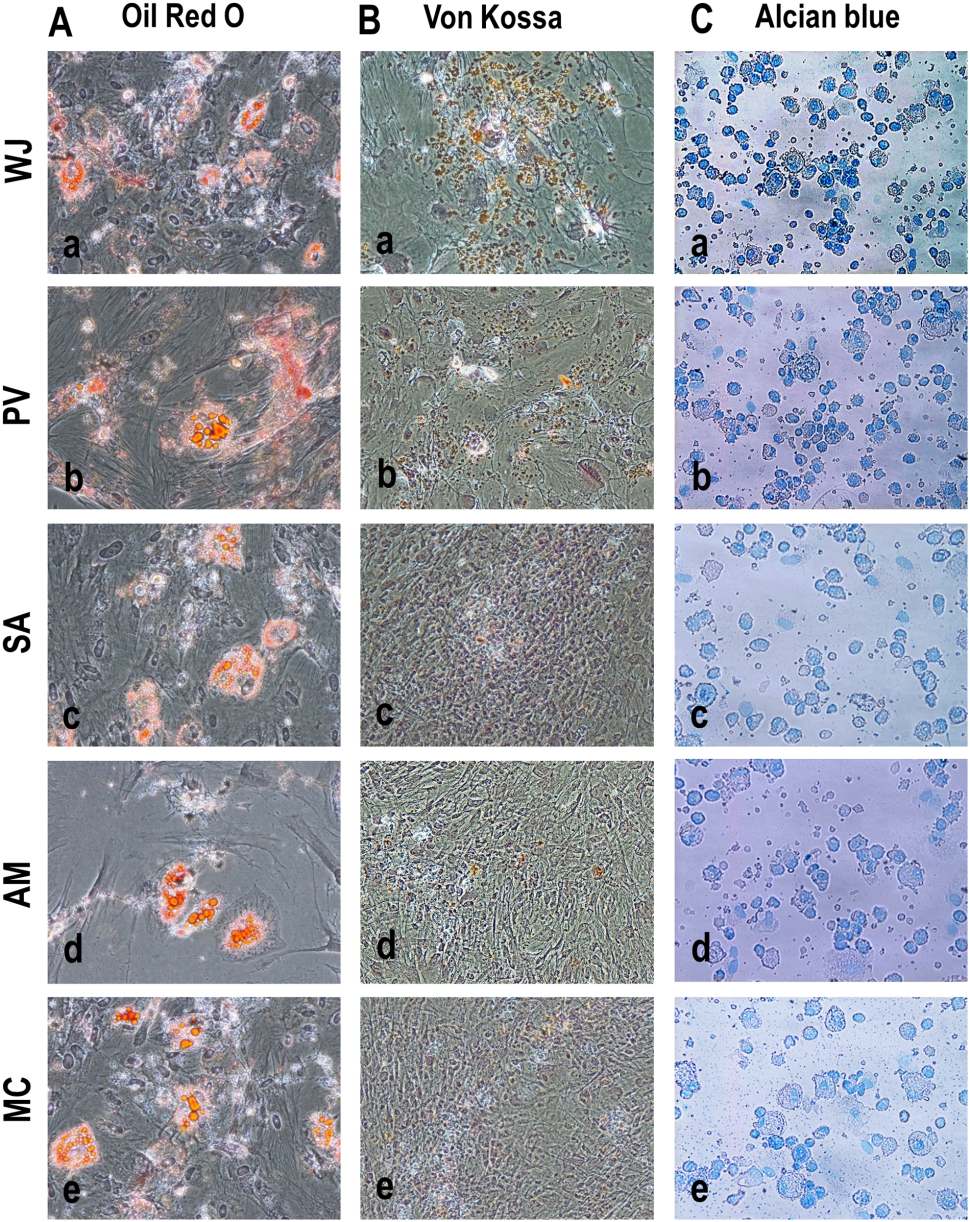
(A-C) Differentiation of cells from WJ, PV, SA, AM and MC into adipogenic, osteogenic and chondrogenic lineages. Cells from WJ, PV, SA, AM and MC stained **(A)** Oil Red O positive for adipocytes, **(B)** Von Kossa positive for osteocytes and **(C)** Alcian blue positive for chondrocytes when exposed to their respective differentiation media for each of the lineages. Adipocyte colonies stained with Oil Red O were similar in number with no differences in staining intensity between WJ, PV, SA, AM and MC. However, Von Kossa and Alcian blue stained cells for osteocytes and chondrocytes were greater in numbers and staining intensities in WJ compared to PV, SA, AM and MC.

The cells from all compartments readily differentiated into adipocyte, osteocyte and chondrocyte lineages (Fig 7). Adipocyte colonies stained with Oil Red O were similar in numbers and there were no significant differences in staining intensity in cells between compartments (Fig 7A). However, the cells from WJ exposed to osteocyte differentiation medium showed the highest number of Von Kossa stained cells and greatest staining intensity of nodules compared to cells from the PV, SA, AM and MC (Fig 7B). Cells from the WJ and PV exposed to the chondrocyte differentiation medium showed higher number of cells that positive for Alcian blue staining compared to cells from SA, AM and MC (Fig 7C).

**Fig 8:**
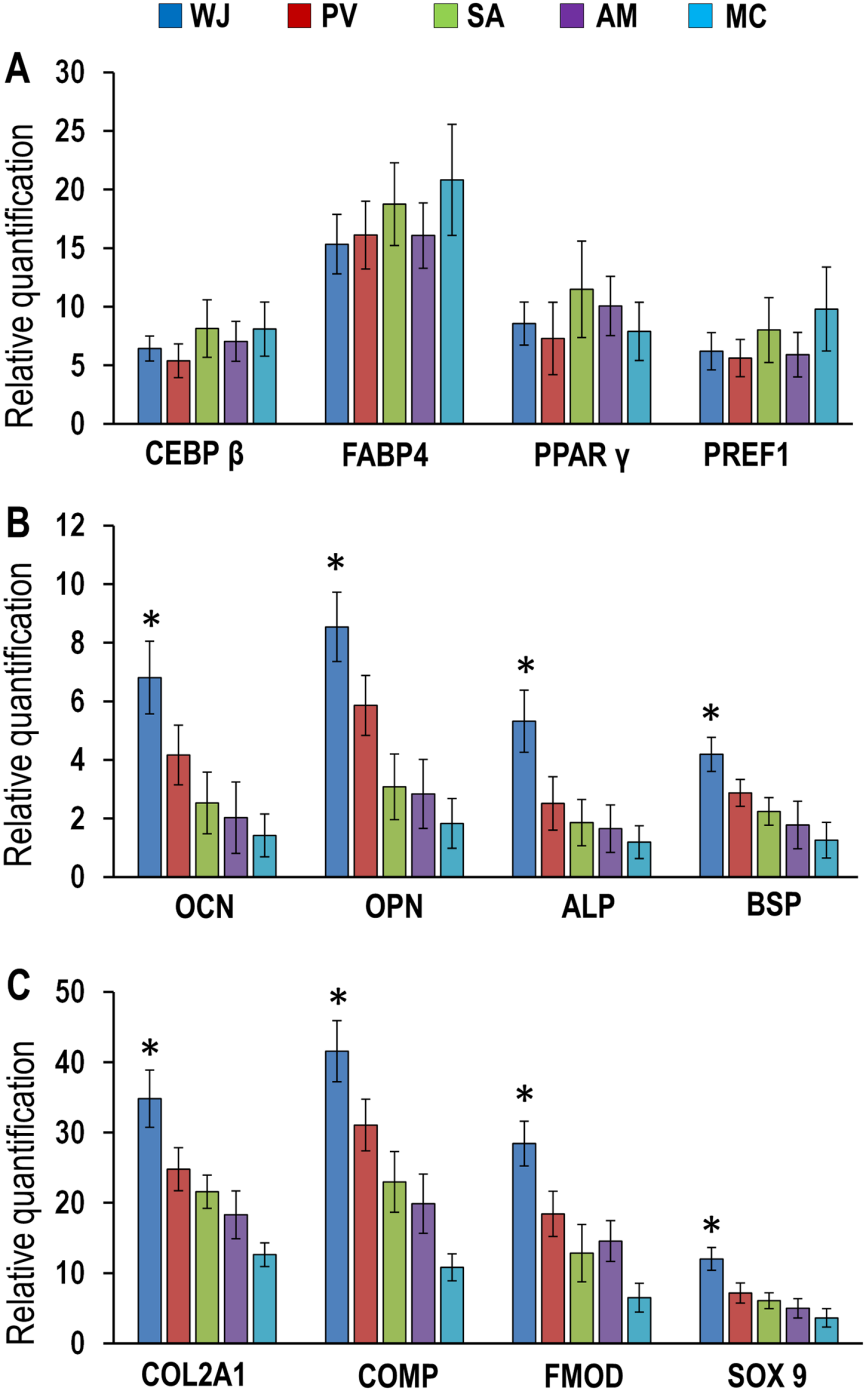
(A-C) Expression levels of adipogenic, osteogenic and chodrogenic genomic markers in differentiated WJ, PV, SA, AM and MC cells. **(A)** The expression levels of the adipogenic-related genes CEBPβ, FABP4, PPARγ and PREF1 were increased for WJ, PV, SA, AM and MC compared to their respective controls with no significant differences between them. **(B)** The expression levels of the osteogenic-related genes osteocalcin (OCN), osteopontin (OPN), alkaline phosphatase (ALP) and bone sialoprotein (BSP) were significantly greater for WJ compared to PV, SA, AM and MC (*p<0.05). **(C)** The expression levels of the chondrogenic-related genes collagen type II (COL2A1), cartilage oligomeric matrix protein (COMP), fibromodulin (FMOD) and sex determining region Y-box 9 (SOX 9) were significantly greater for WJ compared to PV, SA, AM and MC (*p<0.01).

qRT-PCR analysis for adipocyte, osteocyte and chondrocyte genomic markers confirmed that cells from all compartments readily differentiated into these three lineages. There were no significant differences in the expression levels for the adipocyte marker genes CEBPβ, FABP4, PPARγ and PREF-1 in cells between all compartments (Fig 8A). However, the expression levels of the osteocyte marker genes osteocalcin (OCN), osteopontin (OPN), alkaline phosphatase (ALP) and bone sialoprotein (BSP) were significantly greater in cells from WJ (2.93 to 5.39 fold) compared to PV, SA, AM and MC (P<0.05) (Fig 8B). Similarly, the expression levels of the chondrocyte marker genes collagen type II (COL2A1), cartilage oligomeric matrix protein (COMP), fibromodulin (FMOD) and sex determining region Y-box 9 (SOX 9) were significantly greater in cells from WJ (8.40 to 30.75 fold) compared to cells from PV, SA, AM and MC (Fig 8C).

### Degree of differentiation

The comparative scores for the expression levels of the adipocyte, osteocyte and chondrocyte genomic markers showed that the levels were significantly greater for cells in WJ, PV, SA, AM and MC compared to their respective undifferentiated controls (P< 0.05) and there were no significant differences in adipocyte genomic marker levels between WJ, PV, SA, AM and MC (P> 0.05) (Table 2). However, the expression levels of the osteocyte and chondrocyte genomic markers were significantly greater for cells from WJ compared to cells from PV (P<0.05), SA, AM and MC (P<0.01) (Table 2).

**Table 2:** Scoring of adipogenic, osteogenic and chondrogenic differentiation of MSCs derived from various compartments of the UC.

	WJ	PV	SA	AM	MC
Adipogenic differentiation	++	++	++	++	++
Osteogenic differentiation	+++++	+++	++	++	++
Chondrogenic differentiation	+++++	+++	++	++	++

## Discussion

The cells in the SA, AM and PV compartments were tightly attached to each other by an ECM. As such, the SA, AM and PV compartments required manipulation such as enzymatic treatment and prolonged serial cell culture to generate adequate cell numbers for application. The PV region on the other hand produced low cell numbers as it was a small area surrounding the blood vessels. Furthermore, since the SA and AM tightly adhere to each other, any protocol to isolate stem cell populations from each of these compartments runs the risk of cell contamination from the other compartments. In fact, Jeschke et al [20] reported that they had to use a razor blade to separate the SA from AM for their isolation of SA or cord lining MSCs. It has also been reported that the derivation of MSCs from the SA requires several hours as the umbilical cord pieces need to be dissected into small pieces and incubated for 10 to 14 days to be established in culture [7,27,40]. It was also reported that there was the possibility of the cells from SA mixing up with MSCs of the WJ as the two regions were in close proximity and it was thus difficult to exclude cell cross-contamination [20]. Thus the major disadvantages of cells from the SA are that derivation is time-consuming taking at least two working days to process the sample, the tissue often floats in the medium and the resulting cell numbers are low and may not be suitable for rapid and large scale propagation [20,41]. Besides the time taken and labor-intensive nature of isolating MSCs from the SA, AM, PV and MC their manipulation via prolonged serial culture runs the risk of culture-induced genetic changes [42]. More recently, another group re-emphasized that *in vitro* expansion causes dramatic changes in MSC phenotype which has very significant implications for the development of effective therapies [43]. They suggested a ‘one-step’ MSC therapy and discussed the potential cellular and clinical benefits of avoiding too much *in vitro* culture.

In contrast, large numbers of MSCs could be isolated from the WJ with minimal manipulation by simple pipetting in suspension without the need for culture. The results of the present study showed that the WJ compartment of the human UC was the largest in terms of volume and surface area and contained large numbers of fresh live MSCs (4.61± 0.57 x 10^6^/cm) that were of one homogeneous morphological cell type. A normal term UC of 50–60 cm could thus generate large numbers of cells from the WJ that would require only short term culture to generate enough cell numbers for clinical application because of their proliferative nature and short population doubling time thus eliminating the risk of culture-manipulated genetic changes and microbial contamination. Furthermore, their isolation was simple, fast and easy.

In the present study there were no significant differences in the common MSC signature markers (CD29, CD44, CD73, CD90, CD105 and HLA-ABC) between the cells of all compartments (WJ, PV, SA, AM and MC). It has been reported however that the CD24, CD108 and CD40 markers discriminate between MSCs and non-stem cell mesenchymal cell cultures with the increased expression of CD24 and CD108 confirming the presence of MSCs and increased expression of CD40 confirming non-MSC contaminants [36]. The MSCs from the WJ in the present study showed significantly high expression levels of CD24 and CD108 together with very low expression of the fibroblast-specific markers (FAP and FSP) thus confirming that the WJ possessed greater populations of true MSCs.

Increased CD40 expression is typically seen in endothelial cells, smooth muscle cells, fibroblasts and epithelial cells. The cells in the WJ had much lesser CD40+ contaminants (26.12%-27.42%) compared to PV, SA, AM and MC (50.77%-70.22%) further confirming that larger numbers of pure MSC populations were available from the WJ. These percentages of non-MSC contaminants in the present study are consistent with the reports of other workers who showed through flow cytometry studies that only 20% of the umbilical cord matrix cell populations are bona fide MSCs while the rest are stromal cells or normal fibroblasts [44–45]. The results of the present study also showed that the dermal fibroblast marker CD40 (co-stimulatory protein), CD49d (an integrin involved in the homing of cells to an inflammatory site) and CD140b (cell surface receptors tyrosine kinase) were highly expressed in AM, SA and MC cultures suggesting that the stem cell populations in these regions may be mixed with stromal cells or normal fibroblasts [44–45].

MSCs have been isolated from regions around the umbilical blood vessels and referred to as perivascular stem cells [3]. However, in situ histologically stained cross-sections of the human UC did not show an obvious demarcation between PV and WJ. In fact, it has been suggested that all cells from the PV are derived from the WJ region [24]. This may be why some of the results in the present study for the PV were similar to those for WJ. Isolation of cells from the PV compartment has been undertaken because it has been shown that pericytes which belong to the family of MSCs lie around blood vessels [46]. The results of the present study showed that the typical pericyte markers CD146 and CD271 [47] were expressed at significantly higher levels in PV and WJ compared to all other regions. Since there is no clear demarcation between the PV and WJ regions, the intervascular UC matrix may actually encompass both PV and WJ areas thus suggesting that the WJ may contain these pericytes making them the richest region in MSC properties. The major limitations of the PV compartment are that the few cells that could be isolated require long term expansion and may be contaminated with cells from the blood vessel wall on one side and the WJ on the other.

The results of the present study also showed that some stem cell characteristics were different between compartments of the UC with the WJ region being the richest in stem-cell properties. Even though cells from the WJ had high expression levels of the pluripotent markers they did not produce teratomas when injected into immunodeficient SCID mice compared to embryonic (ESCs) or induced pluripotent stem cells (iPSCs) [48–49]. Since the UC (collected 9 months after fertilization) lies in between the 5-day embryo (blastocyst) and adult on the human developmental map, the stem cells isolated from the UC probably start to lose their embryonic pluripotency tumorigenic characteristics and start to acquire multipotent non-tumorigenic MSC characteristics with progressive human development. Interestingly, the telomerase levels of cells from the WJ remained high throughout serial culture compared to all other compartments suggesting that they retain their primitive characteristics in culture for long periods of time. This feature would help cells from the WJ to differentiate into specific lineages more easily during cell-based therapy and allow higher reprogramming efficiency to the embryonic state because of an immature phenotype [50–52].

Although the MSCs derived from each compartment could be differentiated into osteocytes, chondrocytes and adipocytes, there were differences in the degree of differentiation into these lineages. Cells from the WJ showed greater osteogenic and chondrogenic differentiation potential compared to all other compartments within the UC. Several groups have reported that cells from the WJ could also differentiate very efficiently into the neuronal phenotype [53–55].

Some groups for the sake of simplicity dice pieces of UC and grow them as explants (mixed cord, MC) rather than derive cells from individual compartments of the UC [28–29,56–60]. The results of the present study showed that the cell populations from such MC cultures were heterogeneous with mixed islands of cells of different morphologies (epithelioid or fibroblast-like) probably originating from the different compartments. Such heterogeneous cell populations are not ideal for studies or for clinical application as it is not known what influences one morphological cell type has on the other. Majore et al [61] reported that MSCs derived from MC cultures were incapable of osteogenic differentiation even after adding potent osteo-inductive substances such as 1.25-dihydroxy-vitamin D3. The present study confirmed the poor osteogenic and chondrogenic differentiation potential for MSCs derived from MC cultures. Thus, MSCs derived from MC cultures would not be ideal for cell-based therapies due to their heterogeneity, genomic alterations after prolonged manipulation in culture and competition in differentiation along a desired lineage as the cells originate from the various compartments of the UC.

Several groups have also reported other unique advantages of cells from the WJ such as their differentiation into hepatocytes [62], pancreatic-like cells [63], endothelial cells [64], skeletal muscle [65], hyoimmunogenic tolerance to rejection [66–68], non-tumorigenecity [48] and paradoxical anticancer properties [69–78]. It has been hypothesized that cells from the WJ are primitive MSCs that have been trapped in the gelatinous connective tissue matrix of the WJ during migration to and from the placenta during early embryogenesis [79]. The actual role of these cells from the WJ during embryonic and fetal development is not clear although Yang and Chao [80] postulated that their tumoricidal properties may suggest that they act as a natural defence against the migration of cancer cells from the mother to the fetus through the UC because the incidence of malignant cancer cells in the fetus is significantly low in pregnant mothers suffering from ovarian carcinoma, mammary carcinoma, sarcomas or choriocarcinomas of the placenta [80].

Taken together, it appears that MSCs from the WJ are more superior than those from the PV, SA, AM and MC in terms of clinical utility and research value because (i) their isolation is simple, quick and easy to standardize, (i) they have lesser non-stem cell contaminants (iii) they are rich in stemness characteristics, (iv) they can be generated in large numbers with minimal manipulation, (v) they are proliferative and (vi) have broad and efficient differentiation potential. They will thus be stable and attractive candidates for research and future cell-based therapies when derived, propagated and characterized correctly. Our results show that when isolating MSCs from the UC, the WJ should be the preferred compartment, and a standardized method of derivation must be used so as to make meaningful comparisons of data between research groups.
